# Co-Application of Allicin and Chitosan Increases Resistance of *Rosa roxburghii* against Powdery Mildew and Enhances Its Yield and Quality

**DOI:** 10.3390/antibiotics10121449

**Published:** 2021-11-25

**Authors:** Jiaohong Li, Rongyu Li, Cheng Zhang, Zhenxiang Guo, Xiaomao Wu, Huaming An

**Affiliations:** 1 College of Forestry, Guizhou University, Guiyang 550025, China; xhli@gzu.edu.cn; 2 Institute of Crop Protection, College of Agriculture, Guizhou University, Guiyang 550025, China; ryli@gzu.edu.cn (R.L.); 2111816013@stmail.ujs.edu.cn (C.Z.); zxguo3@gzu.edu.cn (Z.G.); 3 Research Center for Fruit Tree Engineering and Technology of Guizhou Province, College of Agriculture, Guizhou University, Guiyang 550025, China

**Keywords:** allicin, chitosan, *Sphaerotheca* sp., antibiotic, *Rosa roxburghii*

## Abstract

Powdery mildew, caused by *Sphaerotheca* sp., annually causes severe losses in yield and quality in *Rosa roxburghii* production areas of southwest China. In this study, the role of the co-application of allicin and chitosan in the resistance of *R. roxburghii* against powdery mildew and its effects on growth, yield and quality of *R. roxburghii* were investigated. The laboratory toxicity test results show that allicin exhibited a superior antifungal activity against *Sphaerotheca* sp. with EC_50_ value of 148.65 mg kg^−1^. In the field, the foliar application of allicin could effectively enhance chitosan against powdery mildew with control efficacy of 85.97% by spraying 5% allicin microemulsion (ME) 100–time liquid + chitosan 100–time liquid, which was significantly (*p* < 0.01) higher than 76.70% of allicin, 70.93% of chitosan and 60.23% of polyoxin. The co-application of allicin and chitosan effectively enhanced the photosynthetic rate and chlorophyll of *R. roxburghii* compared with allicin, chitosan or polyoxin alone. Moreover, allicin used together with chitosan was more effective than allicin or chitosan alone in enhancing *R. roxburghii* plant growth and fruit yield as well as improving *R. roxburghii* fruit quality. This work highlights that the co-application of allicin and chitosan can be used as a green, cost-effective and environmentally friendly alternative strategy to conventional antibiotics for controlling powdery mildew of *R. roxburghii*.

## 1. Introduction

*Rosa roxburghii* Tratt., an edible and medicinal fruit rich in vitamin C, flavonoids, superoxide dismutase (SOD) and various minerals, has high medicinal and nutritional values [1,2,3,4,5]. Recently, the *R. roxburghii* industry has developed rapidly in southwest China, especially in Guizhou Province, where the planting areas reached 170,000 hm^2^ in 2020 [3,6]. Powdery mildew, caused by *Sphaerotheca* sp., is the most serious disease regarding *R. roxburghii* production [1]. In Guizhou Province of southwest China, powdery mildew seriously affects the growth, yield, and quality of *R. roxburghii*, and often causes 30~40% economic losses [7]. Although some chemical fungicides (triadimefon, myclobutanil, azoxystrobin and tebuconazole) [8] and conventional antibiotics (polyoxin and kasugamycin) [9] are frequently used to control powdery mildew, their residuals inevitably affect the environment, wildlife, and human beings [10]. Moreover, these chemicals and antibiotics easily generate resistance to pathogens with the increase in the use frequency [11,12]. Therefore, there is an urgent need to develop an alternative, cost-effective and environmentally friendly control strategy against powdery mildew of *R. roxburghii*.

It is generally believed that natural products are mild and basically harmless compared with chemical fungicides and conventional antibiotics. Although this view is not completely accurate, it has been suggested as one of the reasons for the growing preference of natural products by consumers, and their increasingly popular use in agriculture [13,14]. For instance, Yan et al. [8] reported that 6% ascorbic acid aqueous solutions could induce *R. roxburghii* against powdery mildew with the control effect of 61.45%. Chitosan, a natural resource substance for sustainable agriculture, can be used as a resistance inductor and biofungicide for controlling plant diseases and as a promoter for enhancing plant growth [15,16,17,18,19,20]. In our previous study, the foliar application of 1.0~1.5% chitosan could effectively control powdery mildew of *R. roxburghii* with an inducing control efficacy of 69.30~72.87%, and could notably induce the systemic disease resistance of *R. roxburghii*, as well as reliably enhancing its photosynthesis, growth, yield, and quality [7]. Although chitosan can be used as an effective, safe and economical inductor for controlling powdery mildew, its control effect is still relatively inferior. Thus, natural products enhancing chitosan against powdery mildew of *R. roxburghii* are worthy of further exploration and development.

Allicin, an oxygenated sulfur natural compound, was isolated and identified from garlic in 1944 by Cavallito and Bailey [21]. Since then, allicin has been widely used in agricultural plant protection and medical therapy due to its superior antimicrobial activity and ecofriendly advantage [13,14,22,23,24,25]. Allicin has a prominent reactivity, antioxidant activity and membrane permeability, and can undergo thiol–disulphide exchange reactions with free thiol groups of proteins in microorganisms [13,14,26,27,28]. In agriculture, allicin has been demonstrated to have satisfactory bioactivity against many plant-pathogenic fungi, such as *Plectospherella cucumerina*, *Botrytis cinerea*, *Phytophthora infestans*, *Xanthomonas axonopodis*, *Magnaporthe grisea* and *Alternaria brassicicola* [13,29]. However, to date, there are no documentations available about the application of allicin for controlling powdery mildew of *R. roxburghii* caused by *Sphaerotheca* sp. Moreover, whether allicin can be used as an adjuvant to enhance chitosan against powdery mildew of *R. roxburghii*. is worth further attention.

In this work, the bioactivity of allicin, chitosan and conventional antibiotics against *Sphaerotheca* sp. was firstly determined. Subsequently, the field control efficacy of the co-application of allicin and chitosan for powdery mildew of *R. roxburghii* was evaluated. Moreover, the effects of the co-application of allicin and chitosan on the powdery mildew resistance, growth, yield and quality of *R. roxburghii* were investigated. This study provides a green, cost-effective and environmentally friendly alternative strategy to conventional antibiotics for controlling powdery mildew of *R. roxburghii*.

## 2. Materials and Methods

### 2.1. Fungicides

5% allicin microemulsion (ME) was produced from Ciyuan Biotechnology Co. Ltd. (Xian, China). Chitosan (deacetylation ≥90.00%) was obtained from Huarun Bioengineering Co. Ltd. (Zhenzhou, China). Additionally, 3% polyoxin wettable powder (WP) and 6% kasugamycin WP were provided by Lvdun Biological Products Co. Ltd. (Xian, China).

### 2.2. Field Site

The field experiments were carried out in 2020 in an orchard of *R. roxburghii* with a 7-year-old ‘Guinong 5’ cultivar in Chaxiang village, Gujiao Town, Longli country, Guizhou Province, China (26°54′36″ N, 106°95′13″ E). The planting density of *R. roxburghii* trees was 106 plants per 666.7 m^2^. The annual rainfall, mean temperature, annual sunshine duration, frostless season and mean altitude of field site were about 1100 mm, 13.9 °C, 1265 h, 280 days and 1384 m, respectively. The physical and chemical characteristics of planting soils are shown in Table 1.

### 2.3. In Vitro Toxicity Tests

The *Sphaerotheca* sp. pathogens of powdery mildew on *R. roxburghii* leaves were brushed into sterile water to produce a spore suspension with a concentration of about 100 spores per field of vision under a low power microscope. The healthy young leaves of *R. roxburghii* were washed, and their surface water was air-dried. No pathogen spores were found after microscopic examination. The healthy leaves were made into leaf discs with a diameter of 6 mm using a hole punch. The leaf discs were, respectively, immersed in the five gradient concentration solution of each tested fungicide for 10 s, and then placed in a Petri dish. Ten leaf discs were inoculated in each treatment with four replicates. Powdery mildew spores were inoculated on the front side of leaf discs by spray method, and the medium was sterile water. They were cultured in an artificial climate chamber at 20 °C for 7 days, with 10,000 lx of light intensity, 16 h/d of light duration and 70% relative humidity. The formula for calculating the inhibition rate of pathogens was as Equation (1):

Inhibition rate (%) = 100 × (1 − Diseased leaf counts in treatment/Diseased leaf counts in control dish)(1)

EC_50_ (effective concentration of 50% inhibition rate) values were calculated statistically by a SPSS 18.0 software (SPSS Inc., Chicago, IL, USA).

### 2.4. Field Control Experiment of Powdery Mildew of R. roxburghii

The control experiment of powdery mildew of *R. roxburghii* was carried out using the foliar spray method. The experimental treatments included 5% allicin ME 100–time dilution liquid + chitosan 100–time dilution liquid, 5% allicin ME 100–time dilution liquid, chitosan 100–time dilution liquid, 3% polyoxin WP 100–time dilution liquid and clear water (control). A total of twenty plots were arranged randomly with four replicates and each plot had nine trees. Five trees on the diagonal of each plot were used for determination. Considering powdery mildew mainly damages the fresh young leaves and stems, as well as flower buds, flowers, and young fruits of *R. roxburghii*, about 1.50 L of fungicide dilution liquid was sprayed on each *R. roxburghii* plant (including leaves, stems, flowers and buds) on 31 March and 29 April 2020.

### 2.5. Investigation of Control Effect of Powdery Mildew of R. roxburghii, and Determination of Its Resistance Parameters, Photosynthetic Rate and Chlorophyll

The control effect of tested fungicides for powdery mildew of *R. roxburghii* was investigated on May 30 in 2020 according to Li et al. [7]. The incidence rate, disease index, and control effect of tested fungicides for powdery mildew of *R. roxburghii* were calculated according to Equations (2)–(4), respectively. The incidence degree: 0 = no incidence, 1 = 1~2 diseased lobules with thin hyphae, 2 = 3~4 diseased lobules with thick hyphae, 3 = 5~6 diseased lobules with dense hyphae, 4 = more than 7 diseased lobules with dense hyphae.

Incidence rate (%) = 100 × Number of diseased leaves/Total number of leaves (2)

Disease index = 100 × ∑ (Disease grade value × Number of leaves within each grade)/(Total number of leaves × the highest grade)(3)

Control effect (%) = 100 × (1 − Disease index of treatment/Disease index of control)(4)

The resistance parameters of *R. roxburghii* leaves, such as proline (Pro), soluble sugar, malonaldehyde (MDA), flavonoid, SOD activity and PPO activity, were determined on 30 May 2020 as described by Zhang et al. [30,31]. The photosynthetic rate (Pn) of leaves in *R. roxburghii* was monitored using a portable LI-6400XT photosynthesis measurement system (LI-COR Inc., Lincoln, NE, USA) at 8:00–10:00 a.m. on 30 May. Chlorophyll content of *R. roxburghii* leaves was determined by a UV-5800PC spectrophotometer at 645 nm (OD_645_) and 663 nm (OD_663_) with an acetone–ethanol (*v*/*v*, 2:1) extraction.

### 2.6. Determination of Yield and Quality of R. roxburghii

Fruits of *R. roxburghii* were randomly collected on 2 September in 2020, and then single fruit weight, yield per plant and quality of each plot were determined. The weighing method was used for determining single fruit weight and fruit yield per plant. Fruit quality of *R. roxburghii*, including vitamin C, soluble solid, soluble sugar, total acidity, soluble protein, flavonoid and SOD activity, were also determined as described by Zhang et al. [30,31].

### 2.7. Statistical Analyses

The mean ± standard deviation (SD) of four replicates were exhibited. SPSS 18.0 was used for analyses. Significant differences were determined by a one-way analysis of variance (ANOVA). Origin 10.0 was used for drawing the chart.

## 3. Results

### 3.1. Toxicity of Allicin and Chitosan against Sphaerotheca sp.

The toxicity of allicin, chitosan, polyoxin and kasugamycin against *Sphaerotheca* sp. is shown in Table 2. The 5% allicin ME treatment exhibited an outstanding toxicity against *Sphaerotheca* sp. of *R. roxburghii* with EC_50_ value of 148.65 mg kg^−1^, which was higher by 2.80- 1.24- and 6.95-fold than chitosan, 3% polyoxin WP and 6% kasugamycin WP, respectively. Although chitosan had a relatively inferior toxicity against *Sphaerotheca* sp., its EC_50_ value was 2.48-fold higher than that of 6% kasugamycin WP. The results here indicate that allicin possessed a superior antimicrobial activity compared to conventional antibiotics including polyoxin and kasugamycin.

### 3.2. Field Control Effect of Allicin and Chitosan against Powdery Mildew of Rosa roxburghii

The field control effect of allicin + chitosan, allicin, chitosan and polyoxin against powdery mildew in *R. roxburghii* are shown in Table 3. Allicin + chitosan, allicin, chitosan and polyoxin significantly (*p* < 0.01) decreased the incidence rate and disease index of powdery mildew of *R. roxburghii*, and allicin + chitosan was the most effective. The control effect of allicin + chitosan against powdery mildew was 85.97%, which was significantly (*p* < 0.01) higher than 76.70% of allicin, 70.93% of chitosan and 60.23% of polyoxin. These results indicate that the co-application of allicin and chitosan effectively controlled powdery mildew of *R. roxburghii*, whose control effect was significantly better than that of allicin, chitosan, or polyoxin alone.

### 3.3. Effects of Allicin and Chitosan on Resistance Parameters of R. roxburghii Leaves

**Figure 1:**
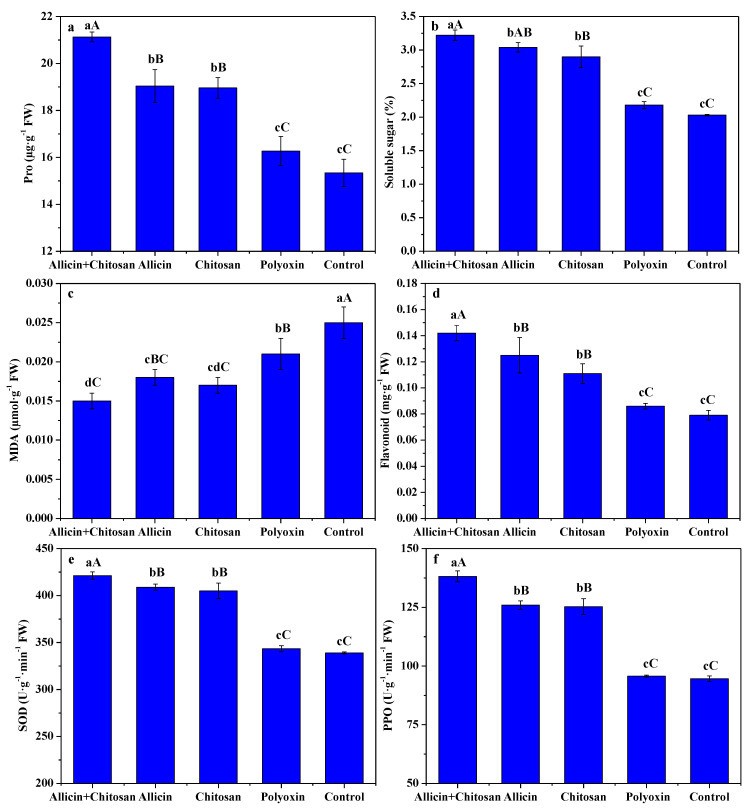
The effects of allicin and chitosan on the Pro (**a**), soluble sugar (**b**), malonaldehyde (**c**), flavonoid (**d**), SOD activity (**e**), and PPO activity (**f**) of leaves in *R. roxburghii*. Values and error bars indicate the mean and SD of three replicates, respectively. Different small letters indicate significant differences at 5% level (*p* < 0.05), and different capital letters indicate significant differences at 1% level (*p* < 0.01).

Figure 1 depicts the effects of allicin + chitosan, allicin, chitosan and polyoxin on the Pro, soluble sugar, MDA, flavonoid, SOD activity and PPO activity of leaves in *R. roxburghii*. Compared to polyoxin or control, allicin + chitosan, allicin and chitosan significantly (*p* < 0.01) increased the contents of proline, soluble sugar, and flavonoid of *R. roxburghii* leaves, and significantly (*p* < 0.01) enhanced their SOD and PPO activities, as well as effectively reducing leaf MDA content. The enhancing effect of allicin + chitosan on Pro, flavonoid, SOD activity and PPO activity of *R. roxburghii* leaves were significantly (*p* < 0.01) higher than that of allicin or chitosan alone. Soluble sugar of *R. roxburghii* leaves treated by allicin + chitosan was significantly higher than that of allicin (*p* < 0.05) or chitosan (*p* < 0.01) alone. MDA of *R. roxburghii* leaves treated by allicin + chitosan was significantly (*p* < 0.05) lower than that of allicin, but had no significant difference to that of chitosan. These results indicate that the co-application of allicin and chitosan effectively improved enhancing or inhibiting effects of allicin or chitosan on the proline, soluble sugar, MDA, flavonoid, SOD activity and PPO activity of leaves in *R. roxburghii*.

### 3.4. Effects of Allicin and Chitosan on Photosynthetic Rate and Chlorophyll Content of R. roxburghii Leaves

**Figure 2:**
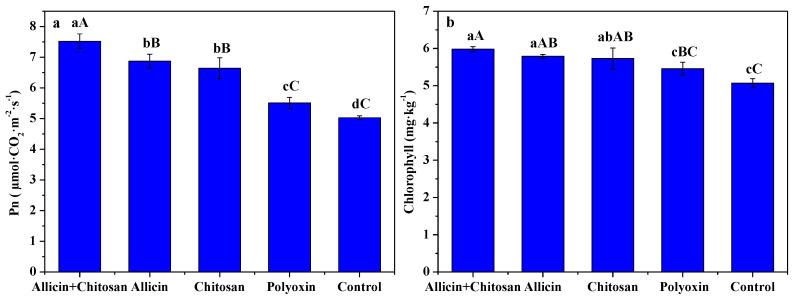
The effects of allicin and chitosan on the photosynthetic rate (**a**) and chlorophyll (**b**) of leaves in *R. roxburghii*. Values and error bars indicate the mean and SD of three replicates, respectively. Different small letters indicate significant differences at 5% level (*p* < 0.05), and different capital letters indicate significant differences at 1% level (*p* < 0.01).

The effects of allicin + chitosan, allicin, chitosan and polyoxin on photosynthetic rate and chlorophyll content in *R. roxburghii* leaves are shown in Figure 2. Compared to polyoxin or control, allicin + chitosan, allicin and chitosan significantly (*p* < 0.01) enhanced the photosynthetic rate of *R. roxburghii* leaves. *R. roxburghii* leaves treated by allicin + chitosan exhibited an excellent photosynthetic rate with 7.52 μmol·CO_2_·m^−2^·s^−1^, which was significantly (*p* < 0.01) higher than 6.87 μmol·CO_2_·m^−2^·s^−1^ of allicin, and 6.64 μmol·CO_2_·m^−2^·s^−1^ of chitosan. Compared to control, allicin + chitosan, allicin and chitosan significantly (*p* < 0.01) increased the chlorophyll of *R. roxburghii* leaves, and there was no significant difference among the three treatments. Compared to polyoxin, allicin + chitosan could significantly (*p* < 0.01) increase the chlorophyll of *R. roxburghii* leaves, while allicin and chitosan could only significantly (*p* < 0.05) increase that of *R. roxburghii* leaves. The results presented here indicate that the co-application of allicin and chitosan effectively promoted leaf chlorophyll of *R. roxburghii*, thereby enhancing its photosynthesis.

### 3.5. Effects of Allicin and Chitosan on Yield and Quality of R. roxburghii

**Figure 3:**
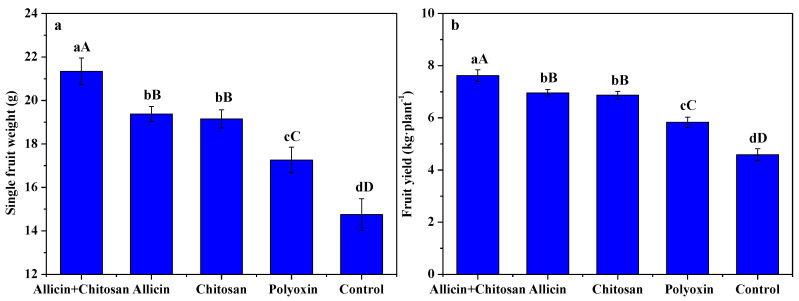
The effects of allicin and chitosan on single fruit weight (**a**) and fruit yield per plant (**b**) of *R. roxburghii*. Values and error bars indicate the mean and SD of three replicates, respectively. Different small letters indicate significant differences at 5% level (*p* < 0.05), and different capital letters indicate significant differences at 1% level (*p* < 0.01).

Figure 3 displays the effects of allicin + chitosan, allicin, chitosan and polyoxin on the single fruit weight and fruit yield per plant of *R. roxburghii*. Compared to polyoxin or control, allicin + chitosan, allicin and chitosan could significantly (*p* < 0.01) enhance the single fruit weight and fruit yield of *R. roxburghii,* and allicin + chitosan was the most efficient. The single fruit weight and fruit yield per plant of *R. roxburghii* treated by allicin + chitosan was 21.34 g and 7.62 kg, which significantly (*p* < 0.01) increased by 10.11% and 9.52%, 11.44% and 10.84%, 23.64% and 30.62%, and 44.68% and 66.11% compared to allicin, chitosan, polyoxin and control, respectively. The results of this work reveal that the co-application of allicin and chitosan effectively promoted fruit growth and yield formation of *R. roxburghii*.

The effects of allicin + chitosan, allicin, chitosan and polyoxin on quality of *R. roxburghii* fruits are displayed in Table 4. Allicin + chitosan, allicin, and chitosan could significantly (*p* < 0.05) increase vitamin C, soluble solid, soluble sugar, total acidity, soluble protein, flavonoid, and SOD activity of *R. roxburghii* fruits compared to polyoxin or control. Vitamin C, soluble solid, soluble sugar, total acidity and SOD activity of *R. roxburghii* fruits treated by allicin + chitosan was significantly (*p* < 0.05) higher than that of allicin or chitosan. In addition, there were no significant (*p* < 0.05) differences between treatments of allicin and chitosan. These findings show that allicin used together with chitosan could effectively improve *R. roxburghii* fruit quality, and allicin and chitosan should have a notably synergistic effect in improving quality of *R. roxburghii* fruits.

## 4. Discussion

Previous findings have demonstrated that allicin could effectively inhibit the growth of *Plectospherella cucumerina*, *Botrytis cinerea*, *Phytophthora infestans*, *Xanthomonas axonopodis*, *Magnaporthe grisea* and *Alternaria brassicicola*, etc. [13,29], and chitosan had antifungal activity against various fungal pathogens [15,16,32,33,34,35]. The results here show that 5% allicin ME displayed outstanding toxicity against *Sphaerotheca* sp., with an EC_50_ value of 148.65 mg kg^−1^, which was 2.80-, 1.24- or 6.95-fold higher than chitosan, 3% polyoxin WP or 6% kasugamycin WP, respectively. This work extended the antimicrobial spectrum of allicin. Although chitosan exhibited a relatively inferior toxicity against *Sphaerotheca* sp., its EC_50_ value was still 2.48-fold higher than that of 6% kasugamycin WP. Moreover, the control effect of powdery mildew of *R. roxburghii* by allicin + chitosan was 85.97%, which was significantly (*p* < 0.01) higher than 76.70% of allicin, 70.93% of chitosan and 60.23% of polyoxin, respectively. Chitosan can trigger plant defense responses by inducing a variety of defense-related reactions [16,34,35,36,37,38]. Our previous results show that the inducing control effect of 1.0~1.5% chitosan against *Sphaerotheca* sp. was 69.30~72.87% [7]. In this study, the co-application of allicin and chitosan significantly (*p* < 0.01) enhanced the control effect of powdery mildew in *R. roxburghii* compared with allicin, chitosan or conventional antibiotic polyoxin alone. This suggests that allicin and chitosan had a notably synergetic effect in the control of powdery mildew of *R. roxburghii*. The effective control effect of allicin + chitosan was probably derived from the superior antimicrobial activity of allicin, as well as the excellent antimicrobial and induced resistance effect of chitosan.

The inducing of disease resistance is an effective agricultural practice for controlling plant diseases [39,40]. Pro and soluble sugar are important regulators of cell permeability, MDA is an important indicator of membrane lipid peroxidation and flavonoid is an important disease-resistant substance, as well as SOD and PPO being defense enzymes associated with plant disease resistance [38,40]. Many studies have also shown that chitosan could induce increases in sugar, Pro, flavonoid, polyphenolics and lignin in the plant and boost its defense enzyme activity, thereby enhancing its disease resistance [16,30,31,32,34,35,36,37,38,39,40]. Our previous results also indicate that the foliar application of 1.0~1.5% chitosan significantly (*p* < 0.01) increased Pro, soluble sugar and flavonoid contents, as well as SOD and POD activities of *R. roxburghii* leaves, and decreased their MDA [7]. The present results show that as compared with polyoxin or control, allicin + chitosan, allicin and chitosan could effectively increase Pro, soluble sugar, and flavonoid of *R. roxburghii* leaves, and enhance their SOD and PPO activities, as well as reduce their MDA, which is consistent with the above studies. Moreover, the enhancing or inhibiting effects of allicin + chitosan on Pro, soluble sugar, flavonoid and MDA contents, as well as SOD and PPO activities of *R. roxburghii* leaves were higher than those of allicin or chitosan alone. These results emphasize that the co-application of allicin and chitosan was more helpful in improving the disease resistance of *R. roxburghii*, and an obviously synergetic effect of allicin and chitosan was available.

Chlorophyll is an essential pigment for plant photosynthesis, and photosynthesis is the physiological basis of plant growth and development. Chitosan can promote plant growth and development by enhancing the photosynthetic rate by increasing chlorophyll content [16]. Our previous results show that foliar application of 0.5~1.5% chitosan effectively enhanced the photosynthetic rate, the content of chlorophyll a, chlorophyll b, and chlorophyll a + b of *R. roxburghii* leaves [7]. In this work, the co-application of allicin and chitosan more effectively promoted the chlorophyll and photosynthetic rate of *R. roxburghii* leaves compared with allicin, chitosan or polyoxin alone. This is closely related to the synergistic effect between allicin protecting plant leaf organs from pathogens and chitosan promoting plant growth. The growth and development of *R. roxburghii* determine its fruit yield and quality. Chitosan can also promote plant growth by activating the auxin and cytokinin signal transduction and gene expression, as well as increasing the nutrient intake [16,41]. Our previous results also indicate that the foliar application of 1.0~1.5% chitosan notably improved yield and quality of *R. roxburghii* fruits [7]. The present results indicate that the co-application of allicin and chitosan effectively enhance *R. roxburghii* fruit growth and yield formation. Moreover, vitamin C, soluble solid, soluble sugar, total acidity and SOD activity of *R. roxburghii* fruits treated by allicin + chitosan was significantly (*p* < 0.05) higher than that of treatments by allicin or chitosan alone. These notable effects were probably derived from their division of labor; allicin can protect *R. roxburghii* from pathogen infection and chitosan can induce the disease resistance of *R. roxburghii*, which guarantee the healthy growth of *R. roxburghii* plants.

At present, increasing attention has been focused on natural products as effective fungicides for controlling plant fungal disease, with high efficacy, nontoxicity and low food safety risks [24,42]. Therefore, natural products as an alternative to traditional antibiotics have been recognized by the public. Allicin, extracted from garlic, is used for daily consumption, and chitosan is a natural, nontoxic substance widely used in food, cosmetics and other fields. Moreover, the safe interval period (from April 29 to September 2, more than 120 days) of *R. roxburghii* was very long. Thus, the food safety risks caused by allicin or chitosan are almost nonexistent. This study highlights that the co-application of allicin and chitosan can be used as a green, cost-effective and environmentally friendly alternative approach to conventional antibiotics for controlling powdery mildew of *R. roxburghii* and enhancing its resistance, growth, yield and quality.

## 5. Conclusions

In conclusion, allicin displayed outstanding antifungal activity against *Sphaerotheca* sp. compared with conventional antibiotics including polyoxin and kasugamycin. The co-application of allicin and chitosan effectively controlled powdery mildew of *R. roxburghii*, and reliably enhanced Pro, soluble sugar and flavonoid contents, SOD and PPO activities in *R. roxburghii* leaves and reduced their MDA contents, as well as notably promoted the photosynthetic rate and chlorophyll contents of *R. roxburghii*. Moreover, the co-application of allicin and chitosan was more effective than allicin or chitosan alone in enhancing growth of *R. roxburghii* plants and improving quality *of R. roxburghii* fruits. This work highlights that the co-application of allicin and chitosan can be used as an ideal alternative to conventional antibiotics for controlling powdery mildew of *R. roxburghii*.

## Figures and Tables

**Table 1:** The physical and chemical characteristics of planting soils of *R. roxburghii*.

Parameters	Content	Parameters	Content
Organic matter	13.17 g·kg^−1^	Exchangeable calcium	18.32 cmol·kg^−1^
Total nitrogen	1.37 g kg^−1^	Exchangeable magnesium	305.37 mg·kg^−1^
Total phosphorus	1.72 g kg^−1^	Available zinc	0.63 mg·kg^−1^
Total potassium	1.11 g kg^−1^	Available iron	6.42 mg·kg^−1^
Available nitrogen	57.43 mg·kg^−1^	Available manganese	15.33 mg·kg^−1^
Available phosphorus	4.21 mg·kg^−1^	Available boron	0.14 mg·kg^−1^
Available potassium	26.75 mg·kg^−1^	pH	6.89

**Table 2:** The toxicity of allicin, chitosan, polyoxin and kasugamycin against *Sphaerotheca* sp.

Treatments	Regression Equation	Determination Coefficient (*R*^2^)	EC_50_ (mg kg^−1^)
5% Allicin ME	*y* = 2.3339 + 1.2274 *x*	0.9626	148.65
Chitosan	*y* = 2.5343 + 0.9413 *x*	0.9748	416.21
3% Polyoxin WP	*y* = 2.9799 + 0.8922 *x*	0.9937	183.68
6% Kasugamycin WP	*y* = 2.4254 + 0.8542 *x*	0.9406	1032.88

*y* and *x* indicate the inhibition rate and fungicide concentration, respectively.

**Table 3:** The control effect of allicin and chitosan against powdery mildew of *R. roxburghii*.

Treatments	Incidence Rate (%)	Disease Index	Control Effect (%)
Allicin + Chitosan	11.00 ± 1.00 ^cC^	2.14 ± 0.18 ^dD^	85.97 ± 1.16 ^aA^
Allicin	14.33 ± 1.53 ^cC^	3.53 ± 0.22 ^cC^	76.70 ± 1.10 ^bB^
Chitosan	16.00 ± 3.61 ^cC^	4.42 ± 0.10 ^cC^	70.93 ± 2.12 ^cB^
Polyoxin	26.67 ± 1.53 ^bB^	6.04 ± 0.19 ^bB^	60.23 ± 4.17 ^dC^
Control	45.67 ± 4.51 ^aA^	15.26 ± 1.12 ^aA^	

Values indicate the mean ± SD of three replicates. Different small letters indicate significant differences at 5% level (*p* < 0.05), and different capital letters indicate significant differences at 1% level (*p* < 0.01).

**Table 4:** The effects of allicin and chitosan on quality of *R. roxburghii* fruits.

Treatments	Vitamin C (mg·g^−1^)	Soluble Solid (%)	Soluble Sugar(%)	Total Acidity (%)	Soluble Protein(%)	Flavonoid ggx(mg·g^−1^)	SOD Activity ggx(U·g^−1^ FW)
Allicin + Chitosan	23.85 ± 0.16 ^a^	12.65 ± 0.08 ^a^	4.21 ± 0.10 ^a^	3.94 ± 0.06 ^a^	15.63 ± 0.47 ^a^	0.127 ± 0.006 ^a^	454.89 ± 2.05 ^a^
Allicin	22.78 ± 0.66 ^b^	12.18 ± 0.15 ^b^	3.92 ± 0.04 ^b^	3.62 ± 0.15 ^b^	14.87 ± 0.72 ^a^	0.119 ± 0.005 ^a^	444.45 ± 4.89 ^b^
Chitosan	22.56 ± 0.59 ^b^	12.12 ± 0.11 ^b^	3.87 ± 0.10 ^b^	3.53 ± 0.14 ^b^	14.59 ± 0.59 ^a^	0.117 ± 0.004 ^a^	441.12 ± 9.72 ^b^
Polyoxin	19.64 ± 0.52 ^c^	11.17 ± 0.13 ^c^	3.26 ± 0.03 ^c^	2.86 ± 0.09 ^c^	13.42 ± 0.61 ^b^	0.108 ± 0.008 ^b^	407.62 ± 5.04 ^c^
Control	17.88 ± 0.61 ^d^	10.35 ± 0.22 ^d^	3.14 ± 0.07 ^c^	2.51 ± 0.14 ^d^	12.65 ± 0.55 ^b^	0.096 ± 0.003 ^c^	376.95 ± 1.49 ^d^

Values indicate the mean ± SD of three replicates. Different small letters indicate significant differences at 5% level (*p* < 0.05).

## Data Availability

The datasets used or analyzed during the current study available from the corresponding author upon reasonable request.
